# Multifunctional graphene metasurface to generate and steer vortex waves

**DOI:** 10.1186/s11671-019-3189-2

**Published:** 2019-11-12

**Authors:** Mengyu Wang, Qingsheng Zeng, Li Deng, Botao Feng, Ping Xu

**Affiliations:** 1 College of Electronic Science and Technology, Shenzhen University, Shenzhen, 518060 China; 2 Key Laboratory of Optoelectronic Devices and Systems of Ministry of Education and Guangdong Province, College of Optoelectronic Engineering, Shenzhen University, Shenzhen, 518060 China; 3 College of Astronautics, Nanjing University of Aeronautics and Astronautics, Nanjing, 211106 China; 4 Beijing Key Laboratory of Network System Architecture and Convergence, Beijing University of Post and Telecommunications, Beijing, 100976 China

**Keywords:** Graphene, Metasruface of multi-functions, Vortex wave

## Abstract

Graphene, an innovated 2D material with atomic thickness, is a very promising candidate and has drawn great attentions in various applications. Graphene metasurface enables dynamic control of various wavefronts, achieving distinguished functionalities. The flexibility of graphene metasurface makes it possible to implement multifunctional devices with ease. In this work, a novel design of multifunctional graphene metasurface, which can combine the functionalities of generating and steering vortex waves, has been proposed. The multifunctional graphene metasurface consists of a large array of graphene reflective unit cells. Each unit cell is controlled independently by its size and external static gate voltage. By scrutinizing the reflective property of the graphene cell, the graphene metasurface is designed to realize multi-functionalities. Simulation results show that vortex wave can be generated and steered. This work can establish a methodology to design multifunctional graphene metasurfaces, and the tunability of graphene opens the gate to the design and fabrication of reconfigurable graphene devices.

## Introduction

Graphene, an innovated 2D innovated material with atomic thickness, is drawing more and more attention in biology, optoelectronics, terahertz communication, etc [1]. In terahertz regime, graphene has better performance than conventional noble metal due to the support of surface plasmon polaritons (SPPs) propagation [2], which makes it a very promising candidate in terahertz technology. Therefore, in recent years, there emerged a great number of graphene-based devices in terahertz and mid-infrared regimes, such as modulators [3–6], detectors [7], absorbers [8, 9], and lasers [10, 11].

It is of great importance to design and fabricate reconfigurable metamaterials to control the behaviour of electromagnetic waves [12, 13]. Therefore, many tuning mechanisms have been realized in various different frequency ranges [14], such as electrically-reconfigurable metamaterials [15], mechanically reconfigurable metamaterials [16], non-linear materials [17], liquid crystals [18], microfluids [19], semiconductor structures [20], and graphene [21]. Graphene, as an innovated material, is a distinguished candidate among them, mainly due to its electric/magnetic controlled conductivity, which enables the design and fabrication of miniaturized controllable devices [14, 22]. Therefore, it has great potential to design reconfigurable metasurface, and many application based on its tunability have been proposed in [23] and [24]. By applying generalized Snell’s law [25, 26], anomalous reflection can be tuned and realized by graphene metasurfaces [27]. These works can pave the way of design and fabrication of tunable terahertz devices.

In telecommunication, orbital angular momentum (OAM) is important to enhance the channel capacity since it can provide infinite state[28, 29]. Three-dimensional metamaterial can be used to generate OAM wave [30]. Metasurface, which can be considered as two dimensional metamaterial, can bring outstanding performance in sub-wavelength thickness. In microwave regime, metasurface have been widely used to design and fabricate devices of subwavelength sizes to generate waves with various polarization and gain properties [31–34]. In terahertz regime, a reflective graphene metasurface has been reported to generate vortex waves with tunability [35]. Graphene metasurface has the flexibility to control the wavefront [36]; therefore, a feasible design, which combines the functionalities of vortex wave generation and anomalous reflection, can be expected to tune the directivity of vortex waves with high precisions.

In this work, based on our previous research on metasurface in micro-nano optics [37–41], we study the mechanism to combine the functionalities of two metasurfaces. A graphene cell is analysed to obtain the relationship between the reflection coefficient and its chemical potential along with its patch size. A full 360 ^∘^ reflection phase range is calibrated as reference to design a graphene metasurface to combine the functionalities of vortex wave generation and anomalous reflection. The combined metasurface is realized by large array of reflective graphene cells. The simulated results show that vortex waves can be generated and steered by a certain angle of reflection.

## Methods

The conductivity of graphene consists of interband and intraband transition. The intraband transition dominates the terahertz and infrared regime, while the interband transition dominates visible optical regime. In terahertz and infrared region, the conductivity can be modelled by Drude model [24],

$$ \sigma(\omega)=\frac{2e^{2}}{\pi\hbar^{2}}k_{B}T\cdot\ln\left[2\cosh\left(\frac{E_{f}}{2k_{B}T}\right)\right]\frac{i}{\omega+i\tau^{-1}},   $$

where *k*_*B*_ is Boltzmann constant, *T* is the temperature, *τ* is the relaxation time, and *E*_*f*_ is Fermi energy.

In this work, the device operates in the terahertz regime, where *E*_*f*_≫*k*_*B*_*T*; hence, the equation can be simplified as

$$ \sigma(\omega)=\frac{e^{2}E_{f}}{\pi\hbar^{2}}\frac{i}{\omega+i\tau^{-1}},   $$

assuming the typical value of room temperature *T*=300*K*, and the relaxation time of graphene *τ*=1 ps. In this work, the Fermi energy *E*_*f*_ is controlled by external static gate voltage. In the simulation, graphene is not modelled as 3D metamaterial blocks but 2D surface conductive conditions due to the atomic thickness.

Graphene metasurface is composed of large array of graphene cells, which results in collective plasmonic behaviour excited on the surface, realizing extraordinary electromagnetic properties. The frequency is 1.3 THz; thus, due to the slow-wave propagation associated with the plasmonic mode, the resonance can occur at very small sizes, i. e., below *λ*/10 [23, 42]. In order to design the metasurface of graphene cells, a calibration graph of the reflective behaviour of a graphene cell is extracted to study the detailed influence of each parameter in a single graphene cell.

**Fig. 1:**
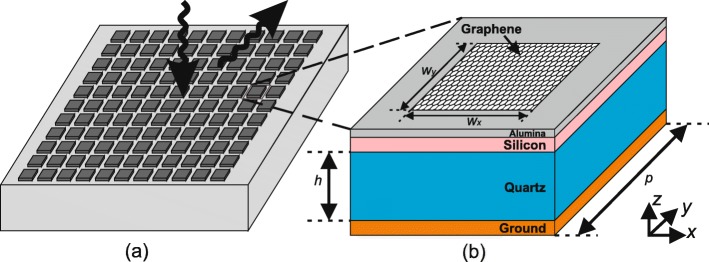
Illustration of graphene metasurface and cell configuration. **a** Schematic of a graphene metasurface, which can steering the incoming electromagnetic waves by anomalous reflection. **b** Configuration of a graphene cell, which consists of multi-layered substrate and a mounted graphene patch of size *w*_*x*_×*w*_*y*_. A static gate voltage is applied between the graphene patch and the silicon layer to control the chemical potential

A typical unit graphene cell, as shown in Fig. 1, is composed of multilayer structure with graphene patch of atomic thickness mounted on the top. The graphene patch with size of *w*_*x*_×*w*_*y*_ is mounted at the center on top of a stack of layered square substrates with side lengths *p* of 14 µm. A quartz substrate (*ε*_*r*_=3.75,tan*δ*=0.0184) of 25-µm thickness is placed on top of the metallic ground layer at the bottom. An external biasing DC voltage is applied between the graphene patch and a polycrystalline silicon layer of 50-nm thickness. A 10-nm-thick Al_2_O_3_ (Alumina, $\epsilon _{r}=8.9, \tan \delta =0.01$) layer is inserted in between as spacer. The chemical potential can be adjusted from 0.01 to 1.0 eV, by controlling the by external biasing DC voltage from 0 to 14.7 V [23, 35]. It should be mentioned that the polycrystalline silicon layer and the Alumina spacer is not modelled in the simulation in this paper and the reasons are as follows. Firstly, a separate 2D simulation, which is much less expensive, is carried out to show that, since the thickness of the polycrystalline silicon layer and the Alumina spacer is much less than the quartz substrate, their influence on the reflective behaviour can be neglected. On the other hand, in the finite element simulations, an extreme amount of elements is required when dealing with adjacent objects with huge difference in sizes. As a result, 3D simulations modelling these two layers will be extremely expensive.

In order to study the reflective properties influenced by *μ*_*c*_ and *w*_*x*_, periodic conditions are assigned in both *x* and *y* directions. The wave impinges normally from top with parallel polarization, i.e., electric field polarized in *x*-direction. Since graphene is equivalent as complex surface conductance condition, only *w*_*x*_ can affect conductance in *x*-direction significantly, while *w*_*y*_ has negligible influence and is fixed as 4 µm in all the simulations in this paper.

**Fig. 2:**
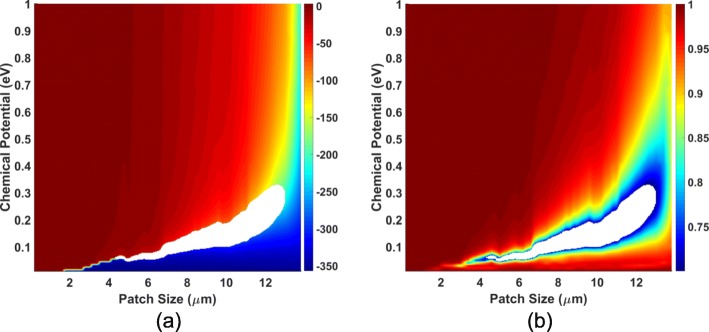
Calibration diagram of reflection coefficients of the graphene cell. The reflection coefficient of the graphene cell influenced by the graphene patch size *w*_*x*_ and the chemical potential *μ*_*c*_, where the region where the magnitude of reflection is smaller than 0.7 is subtracted. **a** phase and **b** magnitude diagram

To scrutinize the influences of patch size and chemical potential, we sweep *w*_*x*_ from 0.2 to 13.8 µm by step of 0.2 µm, and sweep *μ*_*c*_ from 0.01 to 1.00 eV by step of 0.01 eV, and the frequency is fixed at 1.3 THz. The phase and magnitude of *S*_11_ are plotted in Fig. 2, which are called the calibration graphs since the value of *w*_*x*_ and *μ*_*c*_ can be calibrated from them. In order to guarantee the efficiency of the metasurface, the magnitude of the reflection coefficient should be lager that 0.7; thus the unqualified regions are dug out as blank. In the calibration graph, one obtains a full coverage of 360^∘^ which is sufficient to construct graphene metasurfaces.

**Fig. 3:**
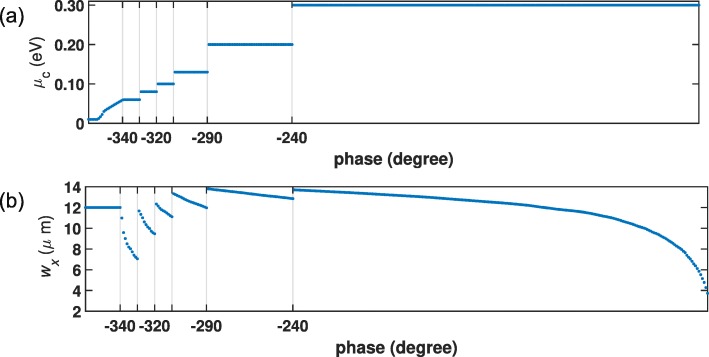
Design diagram of graphene cell. A full 360^∘^ phase coverage achieved by seven groups of combinations of **a** chemical potential and **b** patch size

The phase diagram should be smooth enough to control the phase precisely. In order to design the parameters of graphene cells to achieve full phase coverage from 0^∘^ to 360^∘^, seven combinations of *w*_*x*_ and *μ*_*c*_ are selected, as shown in Fig. 3.

## Results and discussions

To realize various functions, it would be very useful to combine the functionalities of two metasurfaces, or add new functions into another. This methodology will provide versatile way to design new metasurfaces. We combine the functionalities of vortex wave generation and wave deflection by anomalous reflection in this paper.

**Fig. 4:**
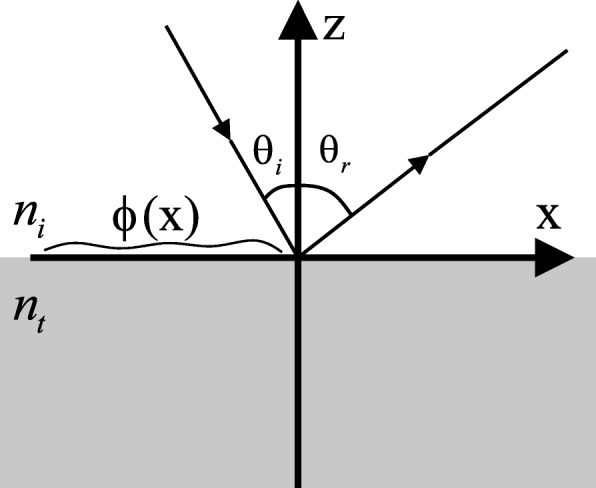
Illustration of generalized law of reflection. An electromagnetic wave impinges from the top with incident angel *θ*_*i*_, while is reflected by *θ*_*r*_ other than *θ*_*i*_, due to phase discontinuity *ϕ*(*x*) along the interface

A generalized methodology is proposed in the following to combine two metasurfaces MS_1_ and MS_2_ into one multifunctional metasurface MS_*t*_. To realize the combination, we start with the generalized law of reflection [25]. As illustrated in Fig. 4, consider a planewave with freespace wavelength *λ* impinges with incident angle *θ*_*i*_, the following equation describes the generalized law of reflection,

1 $$ \sin\theta_{r}-\sin\theta_{i}=\frac{\lambda}{2\pi n_{i}}\frac{\,\mathrm{d}\phi}{\text{dx}},   $$

where *θ*_*r*_ is the angle of reflection, *n*_*i*_ is the the refractive index in upper space, and *ϕ*(*x*) describes the phase discontinuity along the interface.

**Fig. 5:**
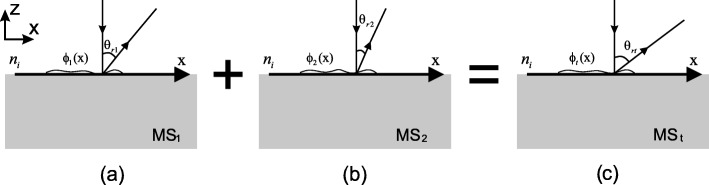
Illustration of combining two metasurfaces into one multifunctional metasurface. In the inset, the electromagnetic waves are impinging normally from upper space with refractive index *n*_*i*_. **a** Metasruface 1 (MS_1_) with phase discontinuity *ϕ*_1_(*x*) and **b** metasurface 2 (MS_2_) with phase discontinuity *ϕ*_2_(*x*) are combined into **c** the desired multifunctional metasurface (MS_*t*_) with phase discontinuity *ϕ*_*t*_(*x*). *θ*_*r*1_(*x*), *θ*_*r*2_(*x*) and *θ*_*rt*_(*x*) are the angles of anomalous reflection along the interfaces of the metasurfaces, respectively, and the relationship *θ*_*rt*_(*x*)=*θ*_*r*1_(*x*)+*θ*_*r*2_(*x*) holds everywhere in MS_*t*_

Consider the simplified case that the wave impinges normally, and the upper space is freespace (*n*_*i*_=1), as shown in Fig. 5, for the first two metasurfaces MS_1_ and MS_2_, Eq. 1 can be further simplified as

2 $$ \frac{\,\mathrm{d} \phi_{m}}{\text{dx}}=\frac{2\pi}{\lambda}\sin\theta_{rm}(x)\quad\quad m=1,2.   $$

To obtain *ϕ*_*t*_ of MS_*t*_, we choose a segment *D*_*x*_ along the interface, and the problem becomes the following: assume in *x*∈*D*_*x*_, holds −*π*/2<*θ*_*r*1_(*x*)+*θ*_*r*2_(*x*)<*π*/2, find *ϕ*_*t*_, s. t. for ∀*x*∈*D*_*x*_, that

3 $$ \begin{aligned} \frac{\,\mathrm{d}\phi_{t}}{\text{dx}}&=\frac{2\pi}{\lambda}\sin\theta_{rt},\quad \text{and}\\ \theta_{rt}(x)&=\theta_{r1}(x)+\theta_{r2}(x). \end{aligned}   $$

It can be derived from Eqs. 2 and 3 that

4 $$ \begin{aligned} \frac{\,\mathrm{d} \phi_{t}}{\text{dx}} &=\frac{2\pi}{\lambda}\sin\theta_{rt} = \frac{2\pi}{\lambda}\sin(\theta_{r1}+\theta_{r2})\\ &=\frac{2\pi}{\lambda}\left(\cos\theta_{r2}\sin\theta_{r1}+\cos\theta_{r1}\sin\theta_{r2}\right)\\ &=\cos\theta_{r2}\frac{\,\mathrm{d} \phi_{1}}{\text{dx}}+\cos\theta_{r1}\frac{\,\mathrm{d} \phi_{2}}{\text{dx}}\\ &=\frac{\,\mathrm{d}}{\text{dx}}\left(\cos\theta_{r2}\phi_{1}+\cos\theta_{r1}\phi_{2}\right)\\ &\quad -\left(\sin\theta_{r2}\frac{\,\mathrm{d} \theta_{r2}}{\text{dx}}\phi_{1}+\sin\theta_{r1}\frac{\,\mathrm{d} \theta_{r1}}{\text{dx}}\phi_{2}\right), \end{aligned}  $$

which leads to

5 $$ \begin{aligned} \phi_{t}(x)=&\cos\theta_{r2}\phi_{1}(x)+\cos\theta_{r1}\phi_{2}(x)\\ &-\int_{D_{x}}\left(\sin\theta_{r2}\frac{\,\mathrm{d} \theta_{r2}}{\text{dx}}\phi_{1}+\sin\theta_{r1}\frac{\,\mathrm{d} \theta_{r1}}{\text{dx}}\phi_{2}\right)\text{dx}, \end{aligned}   $$

where the integration term calculates the contribution of the variance of *θ*_*ri*_(*x*) and can mostly be calculated numerically. Equation 5 plays a vital role to combine the functionalities of two metasurfaces.

Furthermore, if the steering angle is constant, the integration term in Eq. 6 vanishes. Equation 5 can be significantly simplified as

6 $$ \phi_{t}(x)=\cos\theta_{r2}\phi_{1}(x)+\cos\theta_{r1}\phi_{2}(x)+C.   $$

This is the governing equation to combine metasurfaces, and the phase distribution can be calculated to combine vortex wave generation and anomalous reflection.

In this paper, MS_1_ is the metasurface that generates vortex waves, while MS_2_ is the metasurface that steers the waves.

As illustrated in [35], vortex waves with mode *l* can be generated by a plate of *N* sectors with successive increment of phase shift. The phase shift of the *n*th sector *ϕ*_*n*_ can be calculated as *ϕ*_*n*_=*ϕ*_0_+2*πnl*/*N*, where *ϕ*_0_ is the phase shift of the initial sector. Moreover, in order to generate vortex wave, it should be satisfied that −*N*/2<*l*<*N*/2. Therefore, *N*=4 is sufficient to generate modes *l*=0, ±1.

**Fig. 6:**
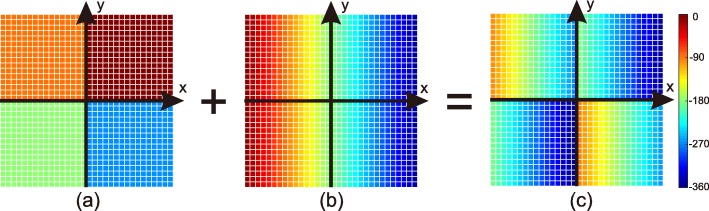
Illustration of combination of phase discontinuity functions. **a** *ϕ*_1_, phase discontinuity distribution of MS_1_, which generates vortex electromagnetic wave with *l*=1. **b** *ϕ*_2_, phase discontinuity distribution of MS_2_, which results in anomalous reflection. **c** Combined phase discontinuity distribution of the MS_*t*_ calculated by Eq. 6

To generate vortex wave with *l*=1, the plate is subdivided into four sectors as shown in Fig. 6a. The phase condition *ϕ*_1_(*x*,*y*) is a piece-wise constant function that decreases by 90^∘^ through sectors, counterclockwisely.

7 $$ \phi_{1}(x,y)=\left\{ \begin{aligned} &0^{\circ} &\quad & x\geq 0, y\geq 0\\ &-90^{\circ} &\quad & x<0, y\geq 0\\ &-180^{\circ} &\quad & x<0, y<0 \\ &-270^{\circ} &\quad & x\geq0, y<0 \end{aligned} \right.   $$

When *x*-polarized wave is impinging normally from above, vortex wave with *l*=1 will be reflected. It should be noted that the wave is reflected vertically; therefore, the deflection angle is 0^∘^, i.e., *θ*_*r*1_(*x*)=0^∘^.

To generate anomalous reflection with deflection angle *θ*_*r*_, Eq. 1 is applied. As illustrated in Fig. 4, when wave is impinging normally in freespace, i.e., *θ*_*i*_=0^∘^ and *n*_*i*_=1, Eq. 1 is reduced to

$$ \phi_{2}(x)=\frac{2\pi\sin\theta_{r}}{\lambda}x+C.   $$

In this work, the deflection angle is set as *θ*_*r*_=30^∘^. From the equation above, by knowing that the period of unit cell is 14 µm, the difference of phase shift between adjacent patches is calculated as 10.9^∘^. The phase distribution is shown in Fig. 6b.

To combine MS_1_ and MS_2_, we take *θ*_*r*1_(*x*)=0^∘^ and *θ*_*r*2_(*x*)=30^∘^ into Eq. 6 and obtain the design formula of MS_*t*_,

$$ \phi_{t}(x)=\frac{\sqrt{3}}{2}\phi_{1}(x)+\phi_{2}(x)+C.  $$

From this formula, one can calculate the phase distribution, which is shown in Fig. 6c. According to Fig. 3, by choosing the chemical potentials *μ*_*c*_ and the patch size *w*_*x*_ of each cell, a 32×32 graphene metasurface is configured. Figure 1a shows the top view of the placement of the graphene cells on the metasurface. One can see that each sector is a 16 ×16 subdomain, consisting 16 columns vertically. And each column consists of 16 identical graphene patches, where a certain combination of *w*_*x*_ and *μ*_*c*_ is assigned.

The plate is excited by an *x*-polarized wave impinging from top. The electric field of the incident wave is normalized, i.e., $ \vec {\mathrm {E}}_{\text {inc}}=\vec {x}$. The simulation was carried out using commercial finite element solver COMSOL Multiphysics 5.2. Graphene has atomic thickness; however, the thickness of the substrates is in micrometre scale. Therefore, computational effort would be tremendous if three-dimensional meshing is applied to graphene patches. Therefore, the thickness of the graphene patches is ignored, and an equivalent two dimensional surface conductivity condition is applied as transition boundary conditions in COMSOL Multiphysics. There are 32×32 patches on the plate, which is subdivided into four sectors. On each sector, there are 16×16 patches controlled independently by their sizes and chemical potentials. The simulation consumed 7.1 million degrees of freedom, which was carried out on a server of 40 ×2.1 GHz threads and 256 GB memory.

**Fig. 7:**
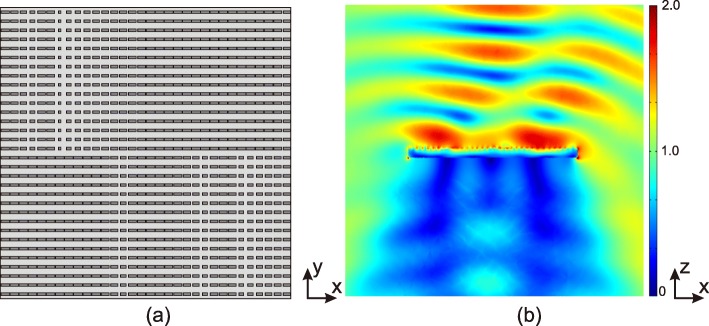
Results of the multifunctional metasurface. **a** Configuration of the plate with graphene reflectarray consisting of 36×36 graphene patches. The widths (*w*_*y*_) of all graphene patch are taken as 4 µm, and values of *w*_*x*_ are selected to realize the phase discontinuity condition as shown in Fig. 6. **b** The magnitude of the electric field of the reflected vortex wave of *l*=1. The incident wave is an *x*-polarized electromagnetic wave with normalized electric field, impinging normally from the top. The wave is deflected by 30^∘^ towards *x*-direction

Figure 7b shows the magnitude of the electric field of the reflected wave normalized by the incident wave. The graphene metasurface generates vortex wave with *l*=1 and deflects by 30 ^∘^ towards *x*-axis.

## Conclusions

In summary, we have studied the design principle of multifunctional graphene metasurfaces. The methodology of combining two metasurfaces is proposed. As an example, a graphene metasurface is designed to combine the functionality of generating vortex wave and steering the waves. Graphene is a two-dimensional atomic thick material, which can dynamically tune the phase condition by applying external gate voltages. Its parameters are scrutinized to calibrate the reflective behaviour of a single graphene cell and obtain coverage of 360^∘^ phase shift. A graphene metasurface consisting of 32×32 unit cells is designed to realize anomalous reflection and generate vortex THz wave simultaneously. Simulation results show that a vortex wave with *l*=1 is generated and steered. Graphene exhibits many extraordinary behaviour in terahertz regime, such as supporting SPP, high efficiency, and tunability; therefore, it is a promising candidate in terahertz technology. This research investigate the approach to combine the functionalities of different metasurfaces implemented by graphene, which opens the gate of dynamically controlled multifunctional metasurfaces in terahertz regime.

## Data Availability

The datasets generated during and/or analyzed during the current study are available from the corresponding authors on reasonable request.

## Abbreviations

OAM: Orbital angular momentum
SPP: Surface plasmon polariton
